# Hygienic

**Published:** 1881-10

**Authors:** 


### HYGIENIC.

It is said that a doctor once stopped on the sidewalk to regard attentively the heaps of unripe fruit, cucumbers and other indigestible merchandise, which was displayed in the shop of a green grocer. He then passed on to the undertaker's, conversed with him confidentially a few minutes, and rubbing their hands in evident satisfaction, they parted in great glee.

Whatever there may be of fiction in this, it is unfortunately too true that physicians in this vicinity have been unusually busy of late in treating those diseases, which are classed under the term dysentery. Undoubtedly many other causes besides unripe fruit produce the same general set of symptoms, but this is one so unusually recognized and one which accounts for so much sickness and so many deaths, especially among children, that we would suggest the rigid observance of the city ordinance which regulates the sale of unripe fruit.

